# Population genetics of an invasive mosquito vector, *Aedes albopictus* in the Northeastern USA

**DOI:** 10.3897/neobiota.78.84986

**Published:** 2022-11-23

**Authors:** Andrea Gloria-Soria, Talya Shragai, Alexander T. Ciota, Todd B. Duval, Barry W. Alto, Ademir J. Martins, Kathleen M. Westby, Kim A. Medley, Isik Unlu, Scott R. Campbell, Malgorzata Kawalkowski, Yoshio Tsuda, Yukiko Higa, Nicholas Indelicato, Paul T. Leisnham, Adalgisa Caccone, Philip M. Armstrong

**Affiliations:** 1 Center for Vector Biology & Zoonotic Diseases, Department of Entomology, The Connecticut Agricultural Experiment Station, 123 Huntington St. New Haven, CT 06504 USA; 2 Department of Ecology and Evolutionary Biology, Yale University, 21 Sachem Street, New Haven, CT 06520-8105 USA; 3 Cornell University, Department of Entomology, 2126 Comstock Hall, Ithaca, NY 14853 USA; 4 Arbovirus Laboratory, Wadsworth Center, New York State Department of Health, 5668 State Farm Road, Slingerlands, NY 12159, USA; 5 Bristol County Mosquito Control Project, 38R Forest Street, Attleboro, MA 02703 USA; 6 University of Florida, IFAS, Department of Entomology and Nematology, Florida Medical Entomology Laboratory, Vero Beach, FL 32962 USA; 7 Instituto Oswaldo Cruz, FIOCRUZ, Rio de Janeiro, RJ, Brazil; 8 Instituto Nacional de Ciência e Tecnologia em Entomologia Molecular, Rio de Janeiro, RJ, Brazil; 9 Tyson Research Center, Washington University in Saint Louis, 6750 Tyson Valley Rd, Eureka, MO 63025 USA; 10 Miami-Dade Mosquito Control Division, 8901 NW 58 St., Miami, FL 33178, USA; 11 Arthropod-Borne Disease Laboratory, Suffolk County Department of Health Services, Yaphank, NY, USA; 12 Division of Vector Control, Suffolk County Department of Health Services, Yaphank, NY, USA; 13 Department of Medical Entomology, National Institute of Infectious Diseases, Toyama 1-23-1, Shinjuku-ku, Tokyo 162-8640, Japan; 14 D.O.T. & I / Highway Division, Mercer County, Ewing, NJ USA; 15 Department of Environmental Science & Technology, University of Maryland, College Park MD 20742 USA

**Keywords:** Asian tiger mosquito, colonization, container-breeder, invasion genetics, propagule pressure, range expansion

## Abstract

The Asian tiger mosquito (*Aedes albopictus*) arrived in the USA in the 1980’s and rapidly spread throughout eastern USA within a decade. The predicted northern edge of its overwintering distribution on the East Coast of the USA roughly falls across New York, Connecticut, and Massachusetts, where the species has been recorded as early as 2000. It is unclear whether *Ae. albopictus* populations have become established and survive the cold winters in these areas or are recolonized every year. We genotyped and analyzed populations of *Ae. albopictus* from the northeast USA using 15 microsatellite markers and compared them with other populations across the country and to representatives of the major global genetic clades to investigate their connectivity and stability. Founder effects or bottlenecks were rare at the northern range of the *Ae. albopictus* distribution in the northeastern USA, with populations displaying high levels of genetic diversity and connectivity along the East Coast. There is no evidence of population turnover in Connecticut during the course of three consecutive years, with consistent genetic structure throughout this period. Overall, these results support the presence of established populations of *Ae. albopictus* in New York, Connecticut, and Massachusetts, successfully overwintering and migrating in large numbers. Given the stability and interconnectedness of these populations, *Ae. albopictus* has the potential to continue to proliferate and expand its range northward under mean warming conditions of climate change. Efforts to control *Ae. albopictus* in these areas should thus focus on vector suppression rather than eradication strategies, as local populations have become firmly established and are expected to reemerge every summer.

## Introduction

The Asian tiger mosquito (*Aedes albopictus*) is a highly invasive species that spread from its native range in East Asia to more than 50 countries on every continent, except Antarctica, during the last 40 years (Sprenger and Wuithiranyagool 1986; Kraemer et al. 2015). The global range expansion and success of this species has been propelled largely by human migration, transportation, and global commerce. *Ae. albopictus* lays desiccation-resistant eggs and develops in artificial water-holding containers, which facilitate its dispersal and establishment in urban and suburban environments (Sprenger and Reiter 1987; Hawley et al. 1987; Parker et al. 2020). Although *Ae. albopictus* feeds opportunistically on a wide range of species (Niebylski et al. 1994; Delatte et al. 2010) it can be an aggressive human biter and a vector of emergent human arboviruses including dengue, chikungunya, and Zika viruses (Metselaar et al. 1980; Gratz 2004; Paupy et al. 2012; Gloria-Soria et al. 2021). This raises the concern that the risk of these arboviruses will increase as this species proliferates and expands its geographic range, as observed in the Indian Ocean Islands, Italy, France, Japan, and Hawaii (Paupy et al. 2009; Grandadam et al. 2011; Rezza 2012).

In the continental USA, *Ae. albopictus* has been detected in 40 states, since the first population was discovered in Houston Texas in 1985 (Sprenger and Wuithiranyagool 1986; Hahn et al. 2017). However, many of these state records could represent transient seasonal introductions rather than established populations. *Aedes albopictus* has become established in southern California and much of the eastern half of the country (Linthicum et al. 2003; Kraemer et al. 2015), with populations continuing to move northward. The northern boundary for overwintering populations has been suggested to be at the isotherm of the coldest month mean temperature of 0 °C based on its distribution in Asia (Nawrocki and Hawley 1987) or isotherms with mean annual temperatures above 11 °C (Kobayashi et al. 2002). This corresponds roughly to southern New England and New York, where *Ae. albopictus* populations emerge annually, with the boundary expected to shift north due to a warming climate (Rochlin et al. 2013). *Ae. albopictus* was first detected in New York (NY) in 2000, in New York City and neighboring Long Island counties in 2003, and is currently spreading north into the Hudson Valley (Kulasekera et al. 2001; Rochlin et al. 2013; Hahn et al. 2016; Kache et al. 2020). In Connecticut (CT), this species was first detected in 2003 and then in 2006 (Andreadis et al. 2005; Andreadis 2009; Armstrong et al. 2017), and has been reported every year since 2010 during continuous statewide mosquito surveillance (Armstrong et al. 2017). Collections occur primarily along the southern margin of CT and successful overwintering of a local population was documented in 2013, during one of the four winters sampled (Armstrong et al. 2017).

We performed population genetic analyses on *Ae. albopictus* collected from NY, CT, and Massachusetts (MA), and compared them to established populations from other USA states and countries to better understand the process of mosquito colonization at the northern expansion front. Collections include mosquitoes sampled from 23 locations along the USA eastern seaboard from Florida to MA, one population from California and temporal collections at four locations in CT spanning three consecutive years. In addition, we include collections from Thailand, Japan, and Brazil as representatives of the major global genetic clusters identified in this species (Kotsakiozi et al. 2017). Here, we characterize the genetic diversity and genetic structure of *Ae. albopictus* populations in the Northeast USA, and evaluate the stability of populations in CT as representatives of the northern edge of *Ae. albopictus* distribution in the USA East Coast; seeking to understand the patterns of *Ae. albopictus* range expansion and establishment in the country. Based on classic invasion theory (Nei et al. 1975; Sakai et al. 2001), we predict low diversity at the northeastern invasion front (CT, NY, MA) relative to the south and the native range, with diversity in the Northeast declining gradually with latitude and evidence of recent bottlenecks consequence of founder events. Furthermore, if these populations have become established we expect stability in their genetic structure over multiple years.

## Methods

### Collections

**Figure 1.**
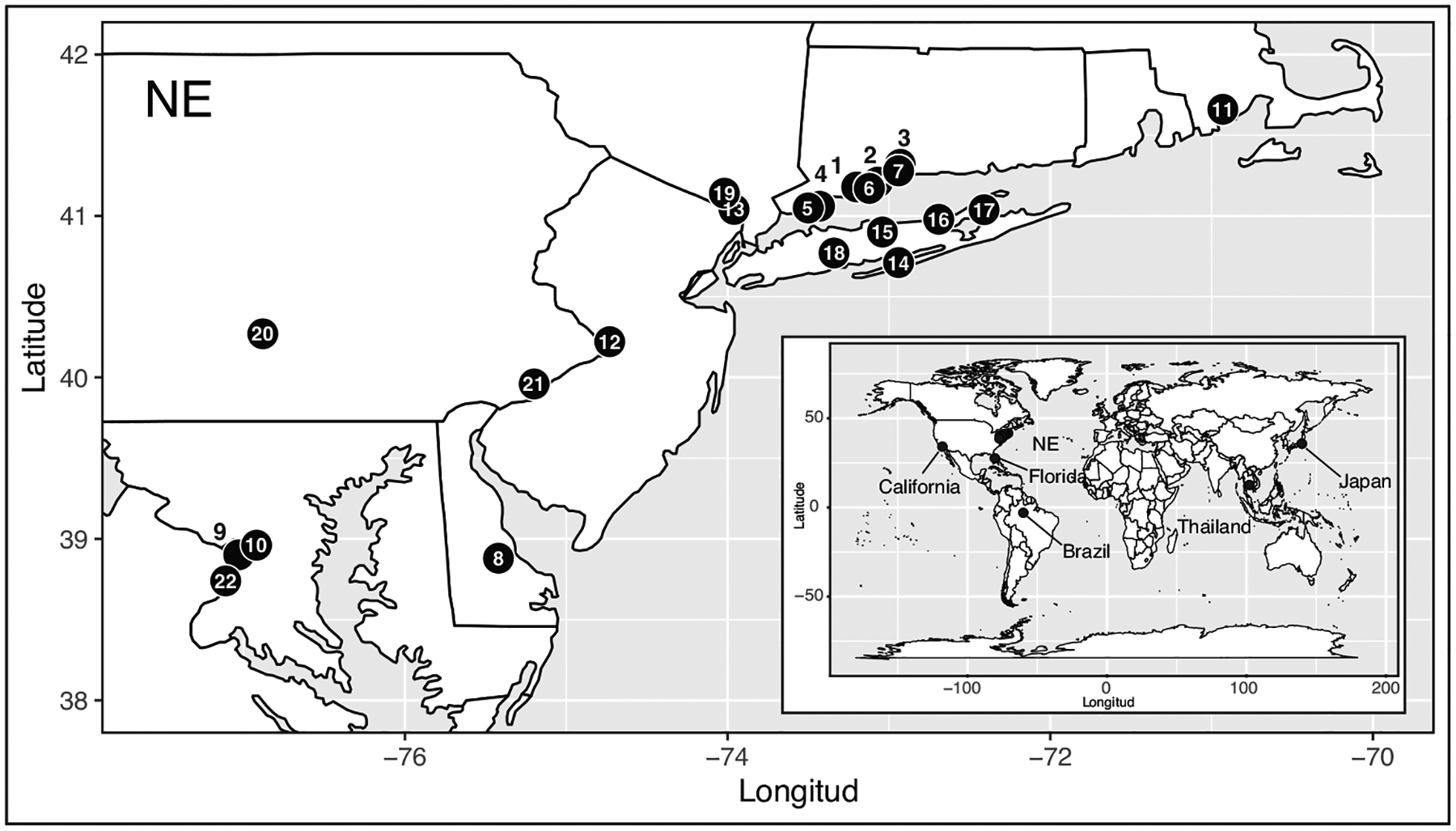
*Aedes albopictus* collection map. Populations of the northeastern USA (NE) are labeled with numbers, corresponding to their ID in Table 1. Outgroups included in this study, representing known genetic clusters are shown in the world map insert in the bottom right corner.

A total of 1,342 *Ae. albopictus* mosquitoes were sent to the Connecticut Agricultural Experiment Station from Departments of Public Health, Mosquito Abatement Districts, and collaborators. All individuals were received as adults directly from the field, with the exception of four sampling sites that were collected as larvae. Larvae from Tappan, NY were reared and underwent one generation in the laboratory, larvae from Fire Island and Spring Valley (NY) underwent 6 generations. Vero Beach samples came from field-collected larvae subsequently reared to adulthood. Samples were received as adults in ethanol and silica gel, with the exception of those of Thailand, Japan, and Brazil which were obtained as DNA aliquots. The samples included 24 locations within the USA (Table 1, Fig. 1 and Suppl. material 1). Temporal samples were collected from Connecticut at four locations every year for three years, with the exception of Norwalk, for which only two years were collected.

### DNA extraction and microsatellite genotyping

Individual mosquitoes were homogenized with a sterile plastic pestle and DNA was extracted following the Qiagen (Hilden, Germany) protocol for purifying total DNA from insects with the Qiagen DNeasy Blood and Tissue Kit (Hilden, Germany), with an additional RNAse A step. Samples were stored at −20 °C until further use. Mosquitoes from Connecticut, which had previously been homogenized in 1 ml of PBS-G media (phosphate buffered saline, 30% heat-inactivated rabbit serum, 0.5% gelatin), were processed following the manufacturers protocol for electrically homogenized samples.

Mosquitoes were genotyped at 15 microsatellite loci, including locus A9 from Porretta et al. (2006), 11 loci from Beebe et al. (2013), and three new loci developed for this study (Suppl. material 2). The AG10, AG01, and AG07 loci were identified during a screen for candidate trinucleotide microsatellite markers using QDD v.3.1. (Meglécz et al. 2014) on *Ae. albopictus* genomic data from Palatini et al. (2020). These new loci successfully genotyped across USA populations in a pilot study and were polymorphic across individuals and populations tested (unpublished data). Polymerase chain reactions (PCR) were conducted as loci combinations (Suppl. material 2) in 10 μl reactions using the Type-it Microsatellite PCR Master Mix (Qiagen; Hilden, Germany) and 200 nM of each forward and reverse primer pairs. Thermocycler conditions were: 95 °C × 5’, 5 touch-down cycles reducing the annealing temperature every cycle by 2 °C from 60 °C to 52 °C (95 °C × 30”, Tm × 30”, 72 °C × 30”), 25× (95 °C × 30”, 50 °C × 30”, 72 °C × 30”), and 60 °C× 30’ for all loci combos, except for loci set #2 (tri25/AG10), for which we used GoTaq DNA polymerase from Promega (Madison, USA). Primer concentrations were the same for the GoTaq reaction with the thermocycler conditions 95 °C × 2’, 5 touch-down cycles reducing Tm every cycle by 2 °C from 61 °C to 53 °C (95 °C × 45”, Tm × 30”, 72 °C × 30”), 25× (95 °C × 45”, 51 °C × 30”, 72 °C × 30”), and 72 °C × 20’.

The resulting products were processed for fragment analysis at the DNA Analysis Facility at Science Hill at Yale University, using GS 500 Liz internal size standard (Applied Biosystems, Waltham MA, USA). Microsatellite alleles were scored using Geneious 11.1.4 (Biomatters Ltd) microsatellite plugin (http://www.geneious.com) using the bins and panels in Suppl. material 3.

Raw allele frequencies are available at VectorBase (www.vectorbase.org), Population Biology Project ID: VBP0000814.

### Genetic diversity

Loci were analyzed for within-population deviations from Hardy-Weinberg equilibrium (HWE) using the Weir and Cockerham (1984) exact test as implemented in Genepop v. 4.7.5 (Raymond and Rousset 1995; Rousset 2008). Null allele frequencies and linkage disequilibrium among pairs of loci (LD) were also estimated with this software. HWE and LD tests were run with 10,000 dememorizations, 1000 batches, and 10,000 iterations per batch. Average observed (*Ho*) and expected (*He*) heterozygosities, and inbreeding coefficients (*G*_*is*_) were estimated for each population in GenoDive 3.04 (Meirmans 2020). Allelic richness (*AR*) was calculated in HP-RARE (Kalinowski 2005), which uses rarefaction to correct for unequal sample sizes (N = 30 genes). Bonferroni correction was applied to the appropriate results to account for multiple testing. A regression analysis in R v. 3.2.2. (R Core Team 2018) was used to evaluate if genetic diversity changed with latitude.

Changes in recent population size were evaluated using Bottleneck v. 1.2.02 (Cornuet and Luikart 1997) under the Infinite Allele Model (IAM) (Maruyama and Fuerst 1985) and the two-phase model (TPM) with a proportion of SMM in the TPM = 0.00 and a variance of the geometric distribution for TPM = 0.36, as recommended by the authors when dealing with microsatellite markers (Cornuet and Luikart 1997). The Wilcoxon sign-rank test (Luikart et al. 1998) was used to determine significance, after Bonferroni multiple test correction.

Effective population size (Ne) was calculated for the temporal collections in CT using NeEstimator (Do et al. 2014) with the Waples (1989) method and three options for computing the standardized variance in allele frequency, *F* [*Fe* (Nei and Tajima 1981); *Fk* (Pollak 1983); and *Fs* (Jorde and Ryman 2007)]; assuming 3 generations per year. *Ne* was also estimated from these populations using a single population sample (as opposed to sampling a population multiple times) with the bias-corrected version of the LD method from Waples and Do (2008). Average *Ne* was estimated using arithmetic and harmonic mean to account for the effect of outliers. Two-sample *Ne* estimates are known to be robust to overlapping generations and can deal with lower levels of polymorphisms (Luikart et al. 2010), but may be affected by changes in allele frequencies occurring during the time lapsed; while single-sample methods are not affected by gene flow and drift but may be biased by overlapping generations and are unable to distinguish from infinite population sizes when not enough polymorphisms are present (Saarman et al. 2017).

Kinship within collections was assessed in ML-Relate (Kalinowski et al. 2006), which uses maximum likelihood estimates of relatedness to discriminate between four common pedigree relationships: unrelated (U), half-siblings (HS), full-siblings (FS), and parent-offspring (PO). The program tests every population for an excess in heterozygosity relative to the observed allelic diversity.

### Population structure

Bayesian clustering analysis was conducted in STRUCTURE v. 2.3 (Pritchard et al. 2000). STRUCTURE identifies genetic clusters and assigns individuals to these clusters with no *a priori* information of sample location. The most likely number of clusters (K) was determined by conducting 20 independent runs from each K = 1 to 8 for the complete dataset, K = 1 to 11 for Japan + America, K = 1 to 10 for the states at the northeastern invasion front (NY, CT, MA), and K = 1 to 11 for the CT temporal dataset. Each run assumed an admixture model and correlated allele frequencies using a burn-in value of 100,000 iterations followed by 500,000 repetitions. The optimal number of K clusters was determined following the guidelines of Prichard et al. (Pritchard et al. 2000) and the Delta K method (Evanno et al. 2005), as implemented by STRUCTURE HARVESTER (Earl and vonHoldt 2012). Results were plotted with the program CLUMPAK (Kopelman et al. 2015) and DISTRUCT v.1.1 (Rosenberg 2004). Discriminant analysis of Principal Components (DAPC) were conducted on allele frequencies using the ADEGENET package (Jombart 2008) in R v. 3.2.2. (R Core Team 2018) from the same datasets analyzed with STRUCTURE, both using pre-defined populations and with the find.clusters command to identify genetic clusters without a-priori information.

Molecular Analysis of Variance was performed in Genodive 3.04 (Meirmans 2020) with 1000 permutations. Pairwise genetic distances (*Fst’*) were calculated in the same software. A geographic distance matrix was produced from geographic coordinates in the Geographic distance matrix generator v. 1.2.3. (Ersts 2016). Correlation between genetic and geographic distance (isolation by distance; IBD) was evaluated for all populations in the Northeast, along I-95 interstate corridor from Virginia (VA) to CT, and across the northeastern invasion front (NY, CT, MA), using a Mantel test and 9999 permutations in the Ade4 package (Dray and Dufour 2007) within R (R Core Team 2018).

## Results

### Genetic diversity

We genotyped a total of 1,342 individual *Ae. albopictus* mosquitoes from 27 geographic locations at 15 microsatellite loci, for an average of 40 individuals per location (Fig. 1, Table 1). Seventy-nine of the 508 possible population-by-locus comparisons (15.55%) deviate from HWE (p < 0.05) after sequential Bonferroni correction. Putative null alleles were inferred at all loci, except for tri20, with average frequencies across populations between 0.02 – 0.22. Linkage disequilibrium is significant in 37 out of the 3,585 locus-by-locus tests (1.03%) after multiple test correction, consistent with the loci being independent.

There is an average of 13.8 ± 6.46 alleles per locus, ranging from 8 to 31, with a mean allele richness (*AR*) across populations of *AR* = 5.13 ± 0.61 (ranging from 4.01 to 7.23; Suppl. material 4). Average observed heterozygosity (*Ho*) is 0.54 ± 0.42, with a lowest value of 0.46 and a highest of 0.66 observed in Brazil and Florida, respectively (Table 1). The average inbreeding coefficient (*G*_*is*_) across populations is 0.17 ± 0.04, with a maximum value of 0.26 in Brazil and a minimum of 0.04 in Florida (Table 1). Regression analysis to establish if genetic diversity decays at the invasion front (higher latitudes) indicates that latitude explains a small part of the variation in *Ho* (adjusted R^2^ = 0.13, F(_1,27_), p = 0.03; Suppl. material 9: fig. S1A), with *Ho* increasing with latitude rather than decreasing. Latitude does not correlate with changes in *AR* (adjusted R^2^ = 0.04, F(_1,27_), p = 0.15; Suppl. material 9: fig. S1B). Genetic diversity at the northern front of the invasion (CT, NY, MA) is no different from that from Japan and Thailand (*Ho*: t_1.02_ = − 1.0411, p = 0.4843; *AR*: t_1.01_ = − 0.9218, p = 0.5248).

Only four populations have evidence of a recent bottleneck. Bottlenecks were inferred for Fire Island and Spring Valley (NY), Mercer County (NJ), and Norwalk (CT), under both the Infinite Allele Model (IAM) (Maruyama and Fuerst 1985) and the two-phase model (TPM) using the Wilcoxon sign-rank test (Cornuet and Luikart 1997) after a Bonferroni multiple test correction (Suppl. material 5). Among them, Fire Island and Spring Valley had been maintained in the laboratory for six generations prior to genotyping, which may explain the bottleneck signature (Table 1).

Local estimates of effective population size across CT using the two-sample method on temporal collections (see Methods) yield mean values of Ne = 94.97 (harmonic mean) and *Ne* = 121.21 (arithmetic mean), ranging from 37.70 to 317.10 (Suppl. material 10: fig. S2A). Single-sample estimations based on LD yield a harmonic mean of Ne = 126.84 and an arithmetic mean of Ne = 2,337.50, ranging from 47.40 to 23,830 (Suppl. material 10: fig. S2B); with the highest value estimated for West Haven (2020) as an outlier.

Analysis of kinship determined that, on average, 1.97% of the pairwise relationships within a population involved first degree pairs (Parent-offspring and full sibling; Suppl. material 6). Tappan NY, Spring Valley NY, and Vero Beach FL have the highest percentage of first-degree pairwise relationships (>5%). Removing first-degree relatives from these populations did not have a major impact in the genetic diversity estimates (t_Ho(4)_ = 0, p = 1; t_Gis(4)_ = 0, p = 1; t_AR(3.98)_ = −0.2964, p = 0.7817), inference of bottlenecks, or the population structure analysis (data not shown).

### Population structure

**Figure 2.**
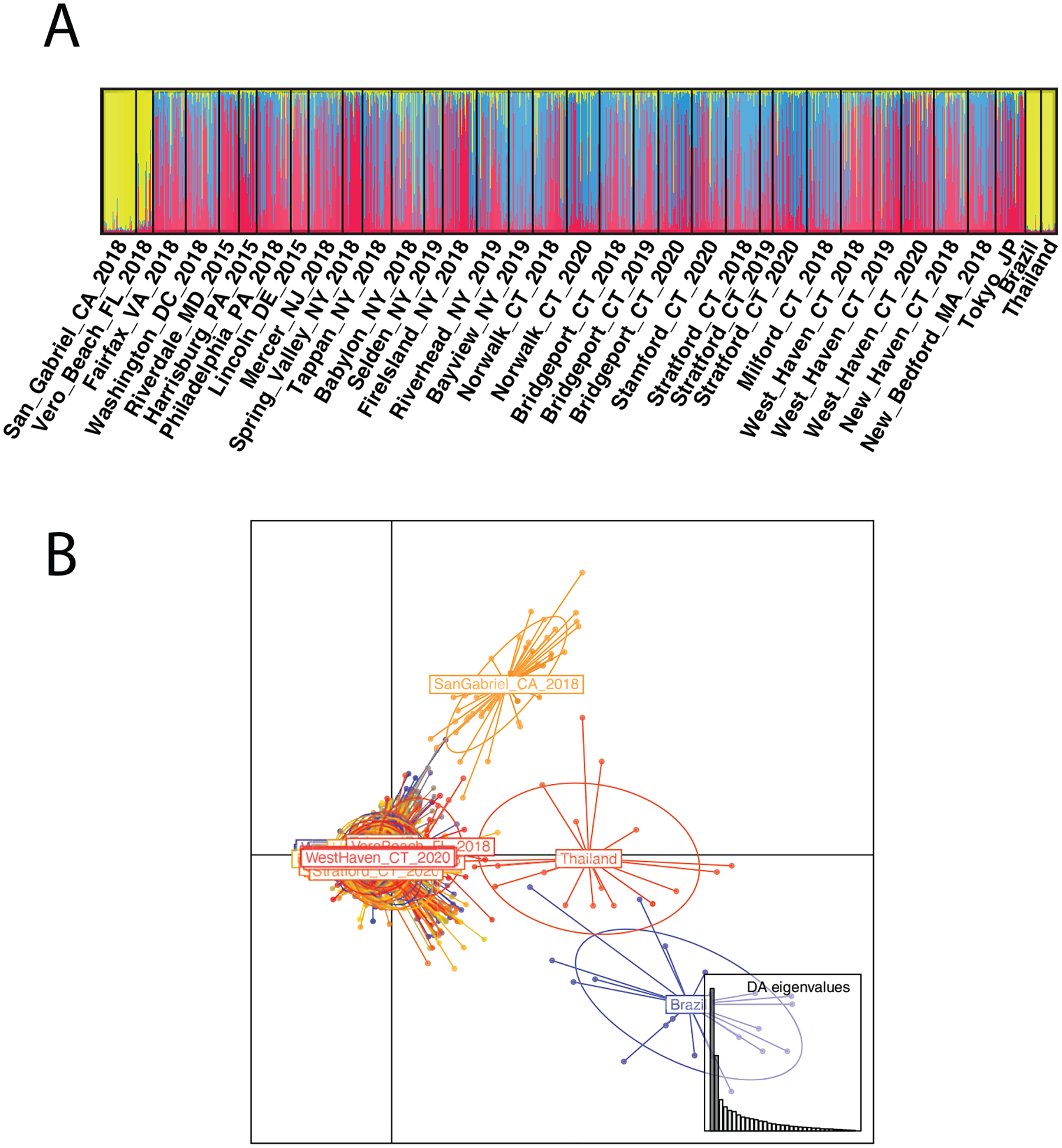
Population structure on the complete *Aedes albopictus* dataset based on 15 microsatellite markers **A** STRUCTURE plot with each individual represented by a vertical bar. The height of each bar is the probability of assignment to each of K = 3 genetic clusters (indicated by different colors) **B** discriminant analysis of principal components (DAPC).

The optimal number of genetic groups inferred from the complete dataset is K = 3, based on Bayesian clustering analysis and the Delta K method (Evanno et al. 2005). The first cluster consists of Florida, California, Brazil, and Thailand while different degrees of admixture between the second and third cluster are observed throughout the rest of the populations analyzed, including Japan and the northeastern USA (Fig. 2A). This grouping is consistent with the DAPC using predefined populations, except that in the DAPC plot Florida is placed within the cluster that includes the northeastern USA (Fig. 2B). No clear genetic structure was detected within the genetic cluster that included Japan and eastern North America, despite a suggested K = 3 using the Delta K method (Suppl. material 11). Incipient population structure is suggested by the clustering analysis of the populations at the northeastern invasion front, with Fire Island and Bayview (NY) showing certain differentiation at K = 3 (Suppl. material 12).

Analysis of Molecular Variance (AMOVA) on the complete dataset indicates that most of the variation can be explained at the individual level, with a lower contribution from the population level (Table 2).

**Figure 3.**
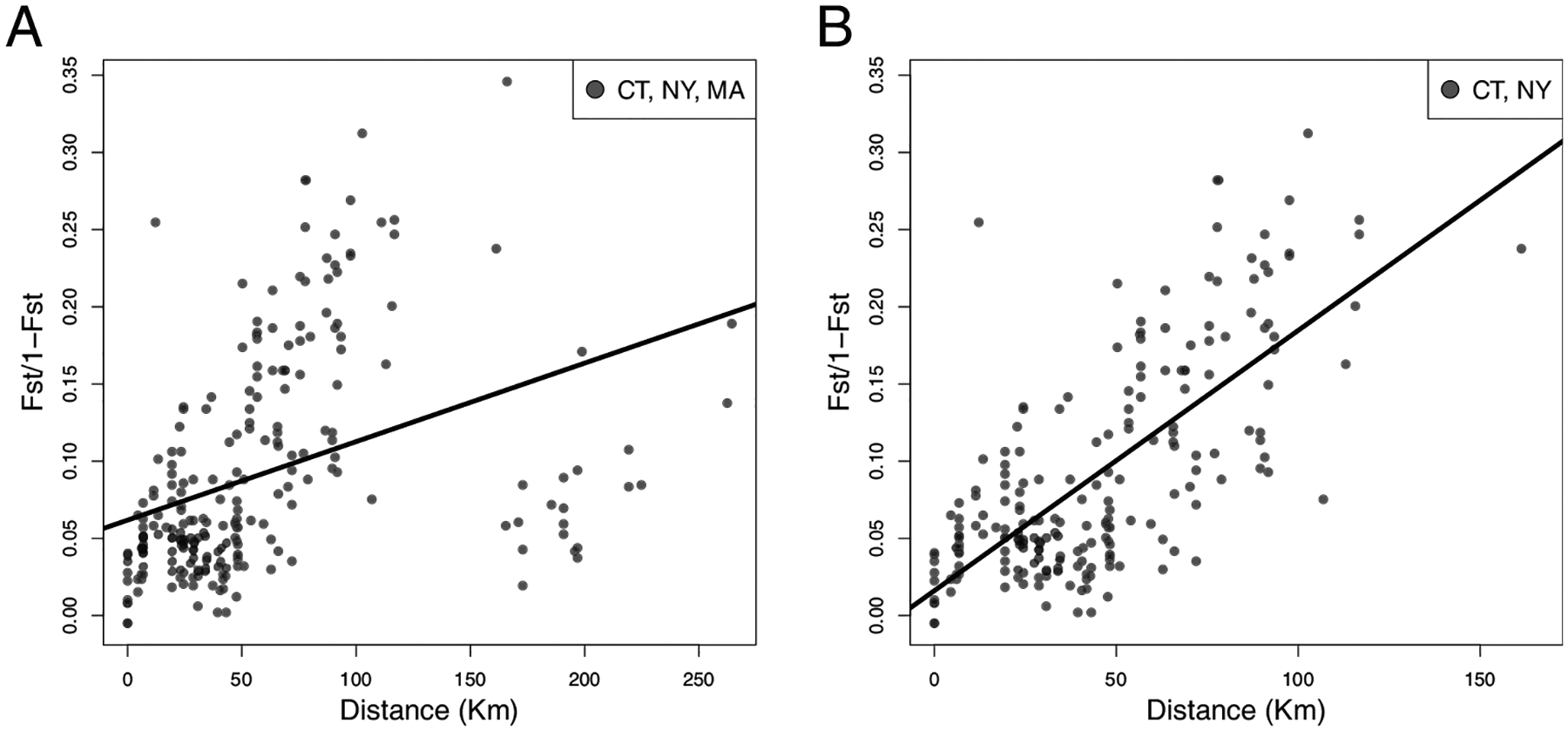
Geographic genetic differentiation (IBD: isolation by distance) across **A** New York, Connecticut, and Massachusetts; and **B** New York and Connecticut. Genetic distance is given as the linearized *F*_*st*_ [*F*_*st*_/(1/*F*_*st*_)] and geographic distance is provided in kilometers (Km). Statistical significance was evaluated using a Mantel test, yielding a significant positive slope only when Massachusetts is excluded (p = 0.072 and p < 0.000 in A and B, respectively).

We then tested for isolation by distance (IBD) throughout the northeastern USA (Virginia, District of Columbia, New Jersey, NY, CT, and MA) to determine whether genetic distance (*F*_*st*_) was correlated with geographic distance (Km) and found no correlation (Mantel statistic = −0.0406, p = 0.4368; Suppl. materials 7, 13). Likewise, there was no IBD in populations located along the I-95 corridor from Virginia to CT (Mantel statistic = 0.088, p = 0.295; Suppl. material 7 and Suppl. material 13), or at the northeastern invasion front: CT, NY, MA (Mantel statistic = 0.382, p = 0.072; Suppl. material 7 and Fig. 3A). However, strong IBD was detected when only NY and CT were analyzed (Mantel statistic = 0.727, p = 0.000; Suppl. material 7 and Fig. 3B).

### Temporal stability

**Figure 4.**
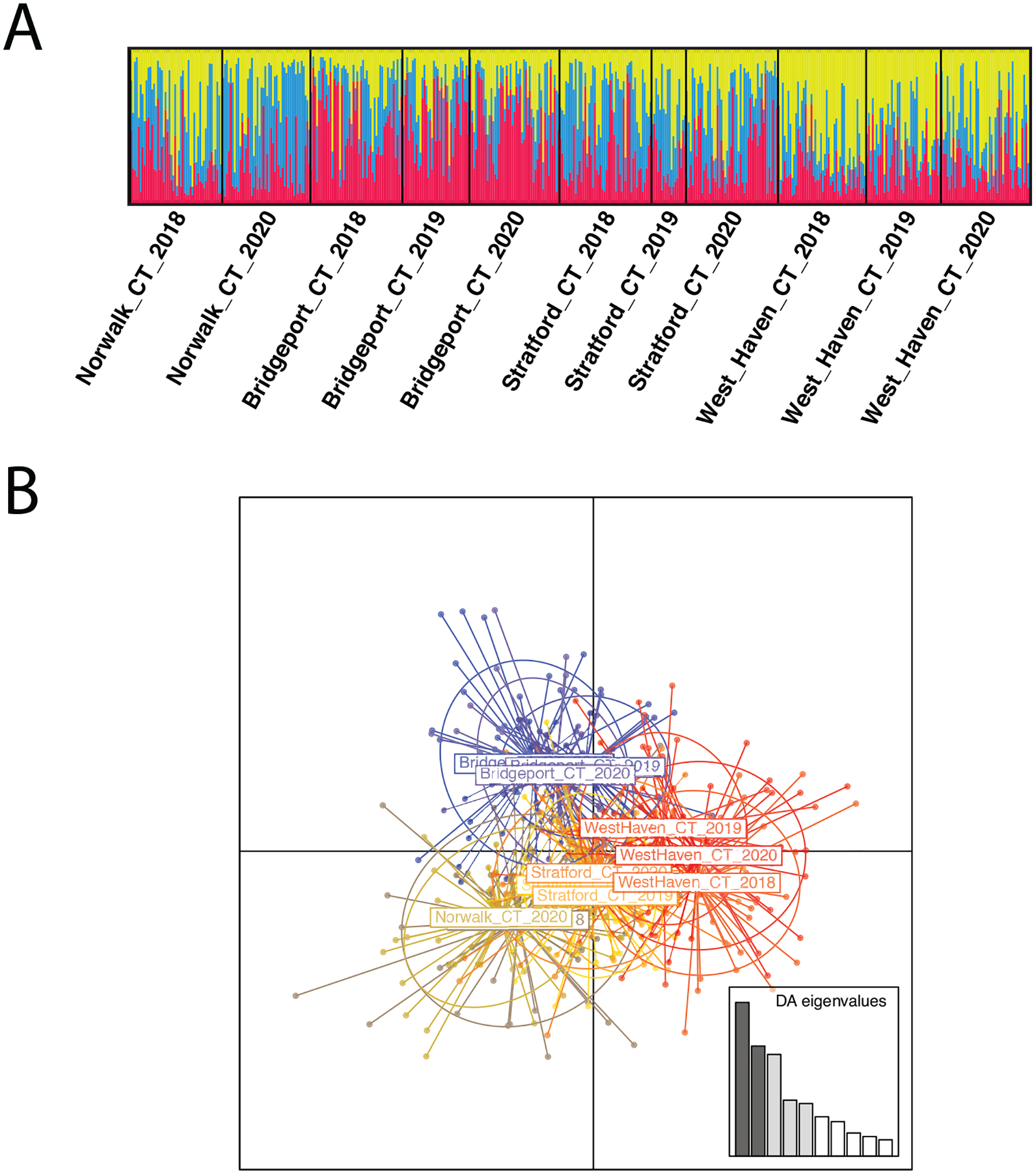
Population structure on *Aedes albopictus* samples from the Connecticut temporal series based on 15 microsatellite markers **A** STRUCTURE plot with each individual represented by a vertical bar. The height of each bar is the probability of assignment to each of K = 3 genetic clusters (indicated by different colors) **B** discriminant analysis of principal components (DAPC). Partially overlapping genetic clusters can be distinguished, grouping temporal collections from the same location.

Bayesian clustering analysis and DAPC across all Connecticut populations indicate weak population structure in CT (Suppl. material 14). Analysis of the temporal series indicates that these population clusters prevail over multiple years, suggesting the development of local populations (Fig. 4). In contrast, there is no support for temporal structure by year of collection (Fig. 4). This result agrees with the AMOVA, with variation mostly explained at the individual and population level rather than by year of collection (Table 3; AMOVA_Time_Points_ p = 0.901). When DAPC was used to infer genetic clusters without population priors, three genetic clusters were inferred (Suppl. material 15). However, these clusters include individuals from all collection points and years (Suppl. material 8), with very few individuals assigned to a third cluster, in agreement with the incipient differentiation suggested by the Bayesian clustering analyses.

## Discussion

We find that *Ae. albopictus* from the northeastern USA are related to *Ae. albopictus* from Japan and harbor high genetic diversity with limited geographic structure. This suggests regional gene flow and a northward invasion driven by a combination of multiple local and long-distance dispersal events that has led to the establishment of northern populations overwintering locally.

Discarded tires are preferred breeding sites for container-inhabiting *Aedes* mosquitoes (Yee 2008) and likely explain how this species entered the country. The USA began importing used tires from Japan in 1968, and by the mid-1970’s most used tires were imported from countries where *Ae. albopictus* was native, mostly from Japan and Taiwan (Sprenger and Reiter 1987). Our results agree with previous work showing that eastern USA populations most likely originated from northern (temperate) East Asia, based on historical records, phenotypic traits (photoperiod sensitivity and cold-hardiness), and genetic markers (Hawley et al. 1987; Kambhampati et al. 1991; Kotsakiozi et al. 2017). We also find that the population in southern California is genetically distinct from those occupying eastern USA, consistent with reports of an introduction of Chinese origin in 2001 and 2011 (Linthicum et al. 2003, Zhong et al. 2013).

Shortly after its initial detection in Texas in 1985 (Moore 1999, Hahn et al 2016), *Ae. albopictus* rapidly spread throughout much of eastern USA. Currently the states of CT, MA, and NY represent the northern limit of the distribution. Classic invasive population genetics predicts that populations at the invasion front would have reduced genetic diversity, consequence of founder effects during the colonization process (Nei et al. 1975; Sakai et al. 2001). We find high genetic diversity (H_o_) at the *Ae. albopictus* northern invasion front, equivalent to that in the native range: Japan and Thailand. Furthermore, evidence of recent bottlenecks (founder effects) was restricted to the two collections from New York that spent 6 generations in the laboratory (Spring Valley and Fire Island), and Mercer County (NJ), and Norwalk (CT). Since bottlenecks are common after laboratory colonization (Gloria-Soria et al. 2019), the bottlenecks detected in Spring Valley and Fire Island are likely the result of the colonization process. A growing number of studies have now demonstrated that the genetic diversity patterns following an invasion event are complex and depend on the size of the propagule (number of individuals invading), frequency of introductions, number of sources, admixture events, or a combination of these (Lockwood et al 2005; Dlugosch and Parker 2008; Facon et al. 2008; Handley et al. 2011; Bock et al. 2015; Jaspers et al. 2021). Different invasion scenarios may result in lower, equal, or higher genetic diversity metrics in the non-native range relative to the native range (Jaspers et al. 2021). High H_o_ values at the invasive range of *Ae. albopictus* have also been reported by others using allozymes (Black et al. 1988), microsatellites (Manni et al. 2017), and genome-wide single nucleotide polymorphisms [SNPs] (Kotsakiozi et al. 2017). The observed genetic diversity in the northeastern USA could be explained by expanding propagules that are subjected to drift and then merge (admixture), or by constant input of alleles that restore the original diversity levels and could possibly exceed them (Lockwood et al. 2005; Facon et al. 2008). In *Ae. japonicus*, another Asian container-breeding mosquito that invaded the USA, merging of two genetic groups was reported in Pennsylvania between 1999/2000 and 2004/2005 and resulted in the loss of the original introduction bottleneck signature and high levels of genetic diversity (Fonseca et al. 2010).

The heterozygosity values observed in *Ae. albopictus* in the Northeast USA are equivalent to those observed in *Ae. aegypti* in the USA (t_17.7_ = 1.027, p = 0.318; Gloria-Soria et al. 2016). Despite this similarity, estimates of inbreeding are an order of magnitude larger in *Ae. albopictus* than in *Ae. aegypti* (Gloria-Soria et al. 2016). High *Ae. albopictus* inbreeding values have been previously reported in the USA using allozymes (Black et al. 1988) and in populations outside *Ae. albopictus* native range with microsatellites (Beebe et al. 2013), and may reflect the local breeding structure of this container mosquito (Black et al. 1988). Alternatively, the increase in homozygosity relative to the expected Hardy-Weinberg equilibrium diagnostic of inbreeding may also be the result of a Wahlund effect or the presence of null alleles, and distinguishing among those mechanisms is not trivial (Barros et al. 2020). We detected putative null alleles at low frequencies (0.02 – 0.22) at all but one of the 15 loci used in this study. Microsatellite null alleles are frequent in insects (Chapuis and Estoup 2007), and in *Ae. albopictus* (Beebe et al. 2013; Manni et al. 2017). Studies have shown that at low frequencies (< 0.20), the presence of null alleles does not affect analyses of genetic diversity and population structure (Dakin and Avise 2004; Chapuis and Estoup 2007; Wei et al. 2019). In 2017 De Meeûs (2018) proposed a statistical test to differentiate the null alleles from a Wahlund effect, based on correlations among F-statistics. Null alleles are expected to increase both *Fis* and *Fst*, creating a strong positive correlation, while a Wahlund effect will move the values in the opposite direction and generate weak or no correlation. We find no correlation between *Gis* and *Gst* (equivalent to *Fis* and *Fst*), indicating that either a Wahlund effect or inbreeding (or both), are most likely responsible for the observed heterozygote deficits rather than null alleles (R^2^ = 0.024, *F*_(1–13)_ = 0.328, p = 0.577). A Wahlund effect arises when genotype proportions are calculated from samples that include individuals belonging to genetically differentiated groups in time or space, for example, subpopulations or cohorts (De Meeûs 2018). It is thus possible that the diversity pattern we observe is the result of substructure within *Ae. albopictus* collections that goes undetected due to the scale of this work. The latter will be consistent with the small neighborhood size estimated for *Ae. albopictus* in Connecticut (*Ne* ~ 100), which is overall lower than those estimated from wild *Ae. aegypti* using 12 microsatellite markers by Saarman et al. (2017).

At the regional scale, *Ae. albopictus* in the northeastern USA is genetically homogeneous. This lack of population structure is congruent with the findings of Kotsakiozi et al. (2017) using ~58,000 genome-wide SNP and likely reflects the demographic features of these species, rather than a lack of marker resolution. One possibility is that being a relatively new invasion there has not been enough time for detectable genetic differentiation to arise. However, fine scale structure is evident in *Ae. aegypti* from California just two years after breeding populations were first detected (Gloria-Soria et al. 2014; Pless et al. 2017). The absence of population structure in *Ae. albopictus* may be better explained by the invasion history of *Ae. albopictus*, spreading faster than *Ae. aegypti* in North America due to its biology and propagule size, and the high connectivity within the region. Consistent with this hypothesis, we detect isolation by distance along CT and NY that does not extend to MA or the rest of the East Coast. This is probably a consequence of the proximity of CT and NY, with gene-flow predominantly occurring via neighboring populations through natural and human-aided dispersal (Handley et al. 2011; Medley et al. 2015). Geographic differentiation within this area is observed and suggests that these populations may already be established and had sufficient time to differentiate.

In Connecticut, *Ae. albopictus* has been recorded every year since 2010 (Armstrong et al. 2017) but it has not been determined whether these populations are present year-round or are reintroduced annually. Unlike its congener *Ae. aegypti*, *Ae. albopictus* is capable of diapausing at the egg stage (Armbruster 2016) and overwintering has been reported in CT after mild winters (Armstrong et al. 2017). If CT was recolonized from the south every year, we would expect that collections from one year will be more similar to each other than between years. We did not find evidence of temporal structure in these collections but rather a weak spatial signature across years, consistent with over-wintering. However, at this point we cannot exclude the possibility that these populations are recolonized by a large influx of individuals from the same sources every year.

## Conclusions

The overall absence of bottlenecks, lack of genetic structure, patterns of isolation by distance, and temporal stability at the northeastern invasive front suggest that *Ae. albopictus* populations in the northeastern USA may already be established as overwintering populations. Furthermore, the high levels of genetic diversity, signatures of inbreeding and small neighborhood sizes suggest that *Ae. albopictus* populations in the northeast USA experience high propagule pressure, probably as the result of multiple, diverse, and frequent invasion sources from southeastern USA populations and possibly from abroad. We suggest that *Ae. albopictus* in eastern USA behave as a metapopulation, in which genetic variation is consistently introduced to the area via human-aided dispersal, and where local genetic drift and selection lead to differentiated small breeding units interconnected across space and time, with admixture through secondary contact further increasing variability.

## Supplementary Material

Supplementary material 01

Supplementary material 02

Supplementary material 03

Supplementary material 04

Supplementary material 06

Supplementary material 05

Supplementary material 07

Supplementary material 08

Supplementary material 09

Supplementary material 10

Supplementary material 12

Supplementary material 11

Supplementary material 15

Supplementary material 14

Supplementary material 13

1

## Figures and Tables

**Table 1.** Population information and genetic diversity based on 15 microsatellite loci.

ID	Location	Year	N	Ho	Hs	Gis	AR
1	Bridgeport, CT, USA	2018	48	0.531	0.664	0.119	5.52
1	Bridgeport, CT, USA	2019	35	0.551	0.657	0.199	5.16
1	Bridgeport, CT, USA	2020	47	0.536	0.642	0.162	5.38
2	Milford, CT, USA	2018	48	0.551	0.667	0.165	5.01
3	New Haven, CT, USA	2018	48	0.591	0.673	0.174	5.07
4	Norwalk, CT, USA	2018	48	0.567	0.678	0.122	5.32
4	Norwalk, CT, USA	2020	46	0.518	0.655	0.164	5.04
5	Stamford, CT, USA	2020	48	0.494	0.637	0.21	4.99
6	Stratford, CT, USA	2018	48	0.573	0.657	0.224	4.95
6	Stratford, CT, USA	2019	18	0.506	0.637	0.128	4.83
6	Stratford, CT, USA	2020	48	0.532	0.646	0.205	4.17
7	West Haven, CT, USA	2018	46	0.564	0.649	0.177	4.92
7	West Haven, CT, USA	2019	39	0.545	0.645	0.132	5.25
7	West Haven, CT, USA	2020	46	0.527	0.662	0.156	5.25
8	Lincoln, DE, USA	2015	25	0.532	0.613	0.204	5.49
9	Washington, DC, USA	2018	47	0.513	0.645	0.132	4.70
10	Riverdale, MD, USA	2015	28	0.494	0.610	0.206	5.20
11	New Bedford, MA, USA	2018	39	0.523	0.633	0.038	5.29
12	Mercer, NJ, USA	2018	48	0.511	0.666	0.191	4.77
13	Tappan, NY, USA *	2018	41	0.531	0.641	0.175	4.85
14	Fire Island, NY, USA *	2018	48	0.537	0.657	0.232	5.18
15	Selden, NY, USA	2019	26	0.556	0.669	0.173	4.40
16	Riverhead, NY, USA	2019	45	0.570	0.684	0.182	4.56
17	Bayview, NY, USA	2019	34	0.530	0.647	0.169	5.46
18	Babylon, NY, USA	2018	46	0.555	0.649	0.168	5.62
19	Spring Valley, NY, USA*	2018	28	0.485	0.597	0.180	4.80
20	Harrisburg, PA, USA	2015	25	0.496	0.655	0.145	5.16
21	Philadelphia, PA, USA	2018	48	0.535	0.625	0.188	4.01
22	Fairfax, VA, USA	2018	46	0.499	0.625	0.243	5.04
-	Vero Beach, FL, USA	2018	24	0.658	0.684	0.145	4.95
-	San Gabriel, CA, USA	2018	47	0.593	0.673	0.201	6.88
-	Manaus, Brazil	2017/18	22	0.459	0.622	0.262	4.88
-	Tokyo, Japan	2017/18	42	0.542	0.647	0.162	4.96
-	Chanthaburi, Thailand	2016	20	0.651	0.740	0.121	7.23

ID: location identifier in Fig. 1. Locations beyond the focus area are shown in the insert of Fig. 1 and were not assigned an ID; N: number of individuals; Ho: observed heterozygosity; Hs: expected heterozygosity; Gis: Inbreeding Coefficient; AR: estimated by rarefaction (N = 30 genes).

* underwent 1–6 generations in laboratory.

**Table 2.** Analysis of Molecular Variance on all populations genotyped for 15 microsatellite loci.

Source of Variation	Nested in	% var	F-stat	F-value	Std.Dev.	P-value
Within Individual	–	0.792	F_it	0.208	0.049	–
Among Individual	Population	0.160	F_is	0.168	0.050	0.001
Among Population	–	0.047	F_st	0.047	0.004	0.001

**Table 3.** Analysis of Molecular Variance on temporal samples from Connecticut genotyped for 15 microsatellite loci.

Source of Variation	Nested in	%var	*F*-stat	*F*-value	Std.Dev.	P-value
Within Individual	–	0.801	*F_it*	0.199	0.044	–
Among Individual	Population	0.183	*F_is*	0.186	0.044	0.001
Among Population	Series_A	0.018	*F_sc*	0.018	0.004	0.001
Among Time points	–	−0.002	*F_ct*	−0.002	0.002	0.901
