# Supplementary figures and images for: Population genetics of an invasive mosquito vector, Aedes albopictus in the Northeastern USA
Source: NeoBiota. Author manuscript; Available in PMC 2023 Jul 5. (PMC10321554; doi:10.3897/neobiota.78.84986)

**A**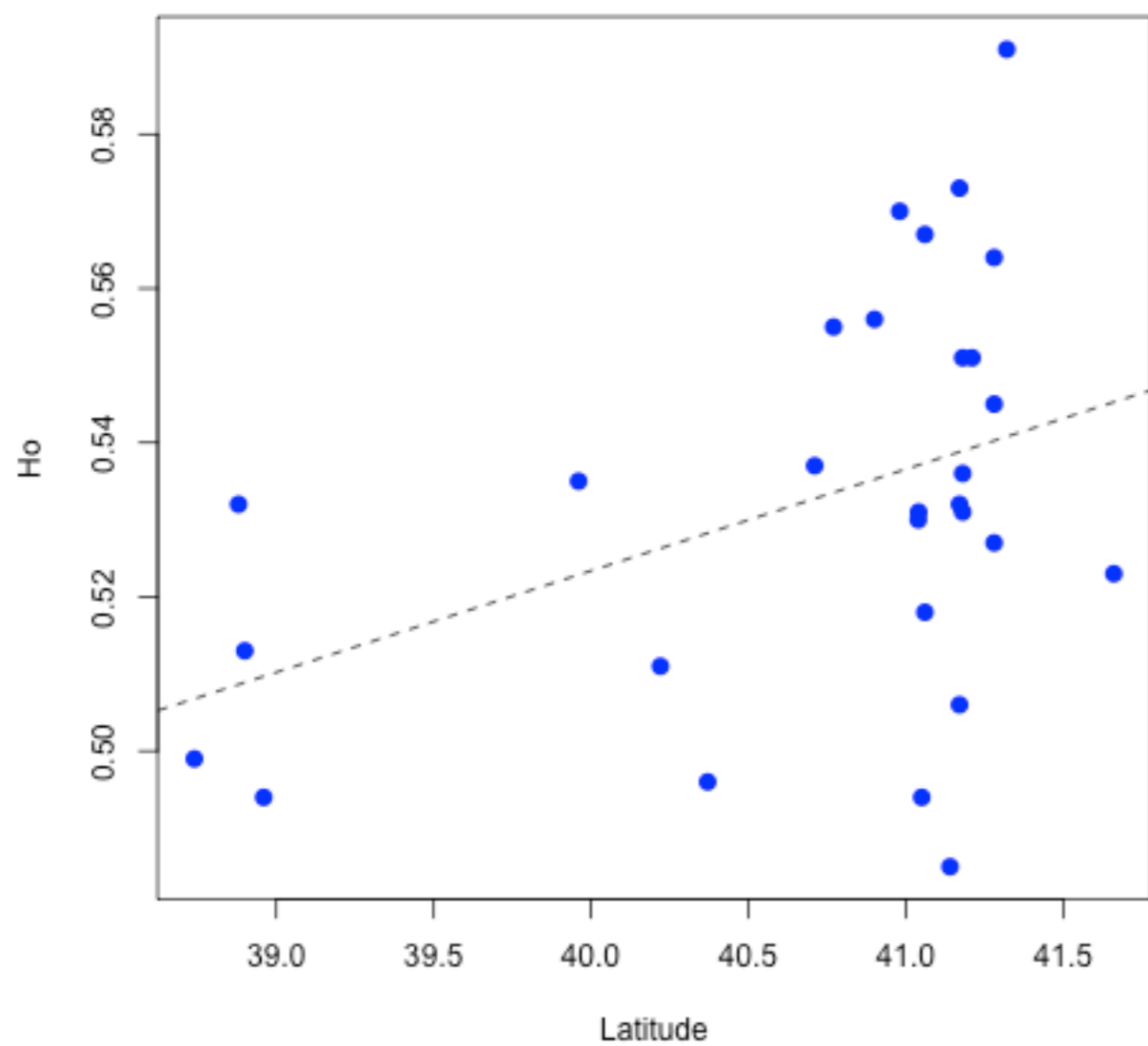**B**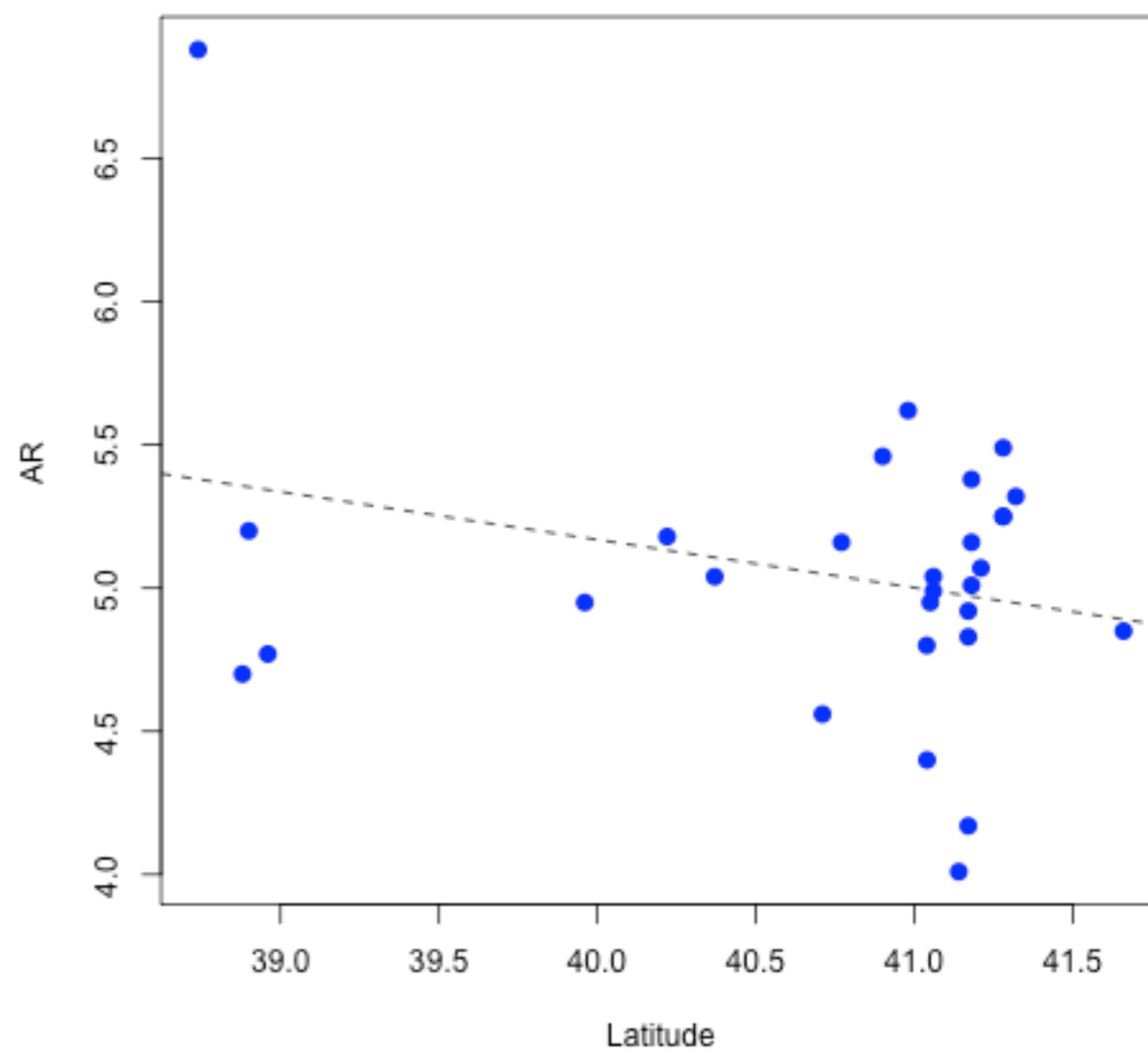

Supplement: Supplementary material 09 [file NIHMS1858977-supplement-Supplementary_material_09.pdf]

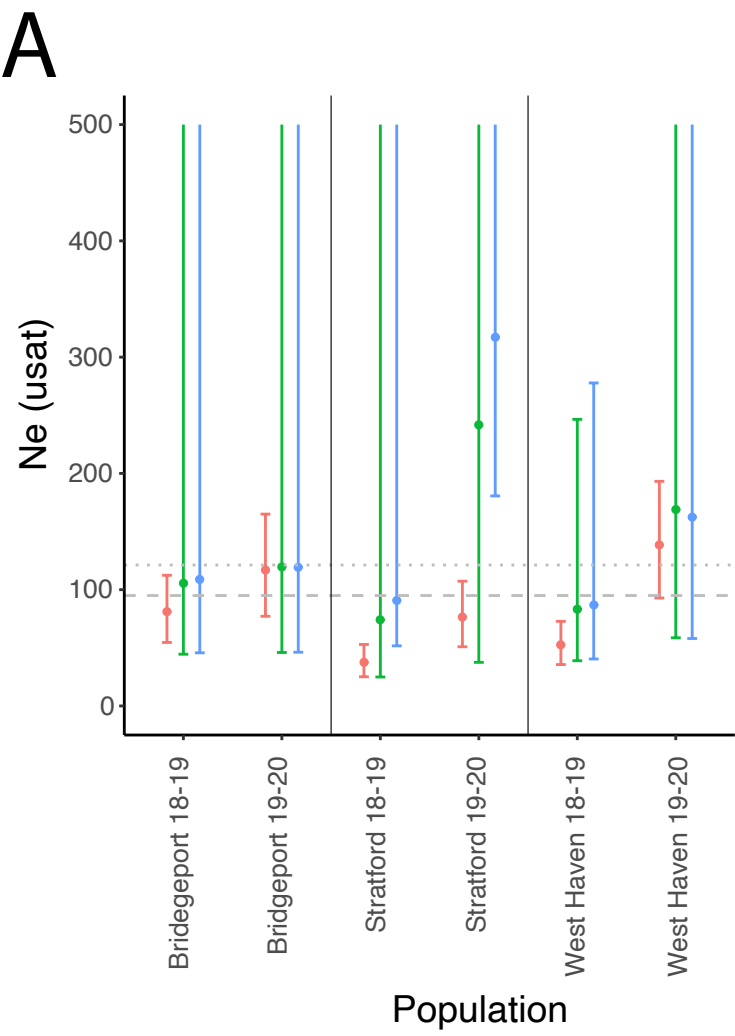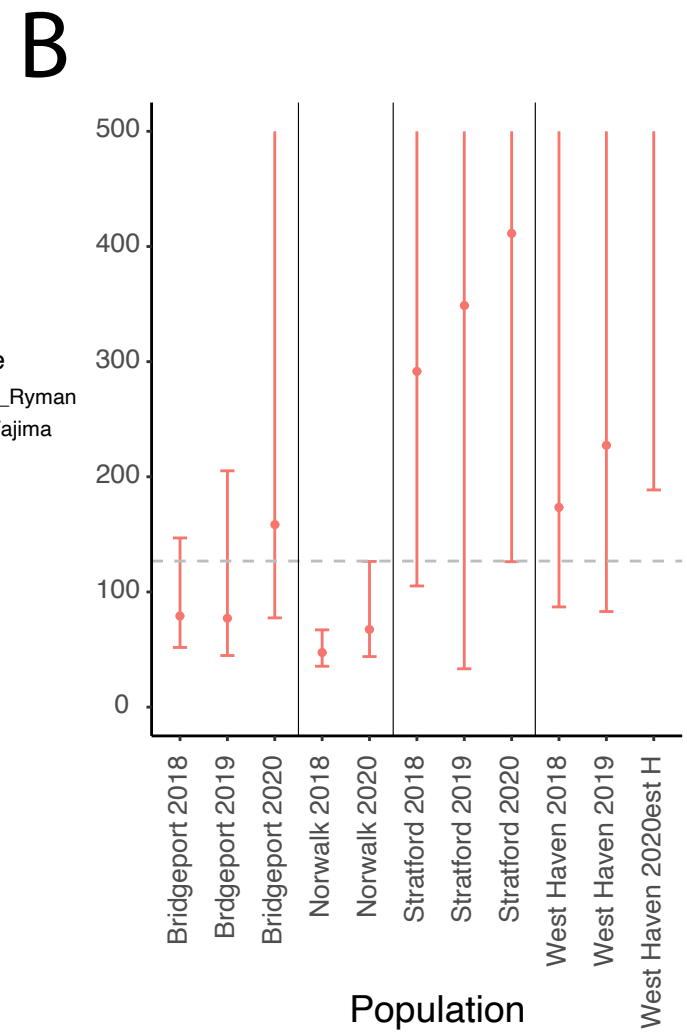

Supplement: Supplementary material 10 [file NIHMS1858977-supplement-Supplementary_material_10.pdf]

A

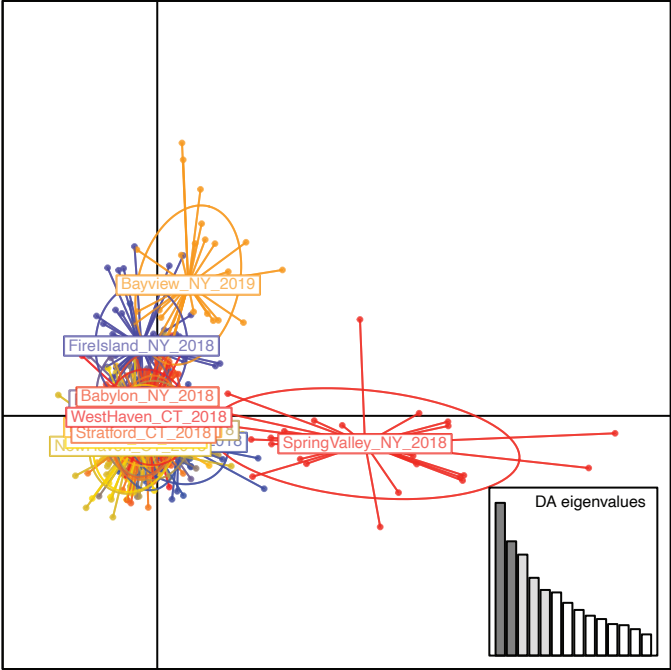

B

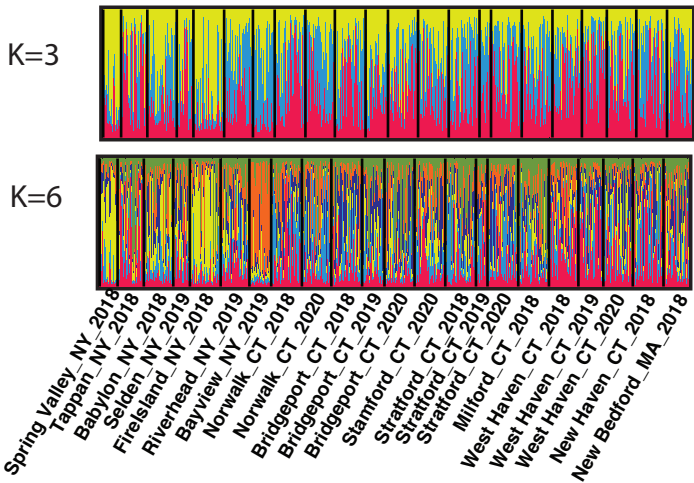

Supplement: Supplementary material 12 [file NIHMS1858977-supplement-Supplementary_material_12.pdf]

A

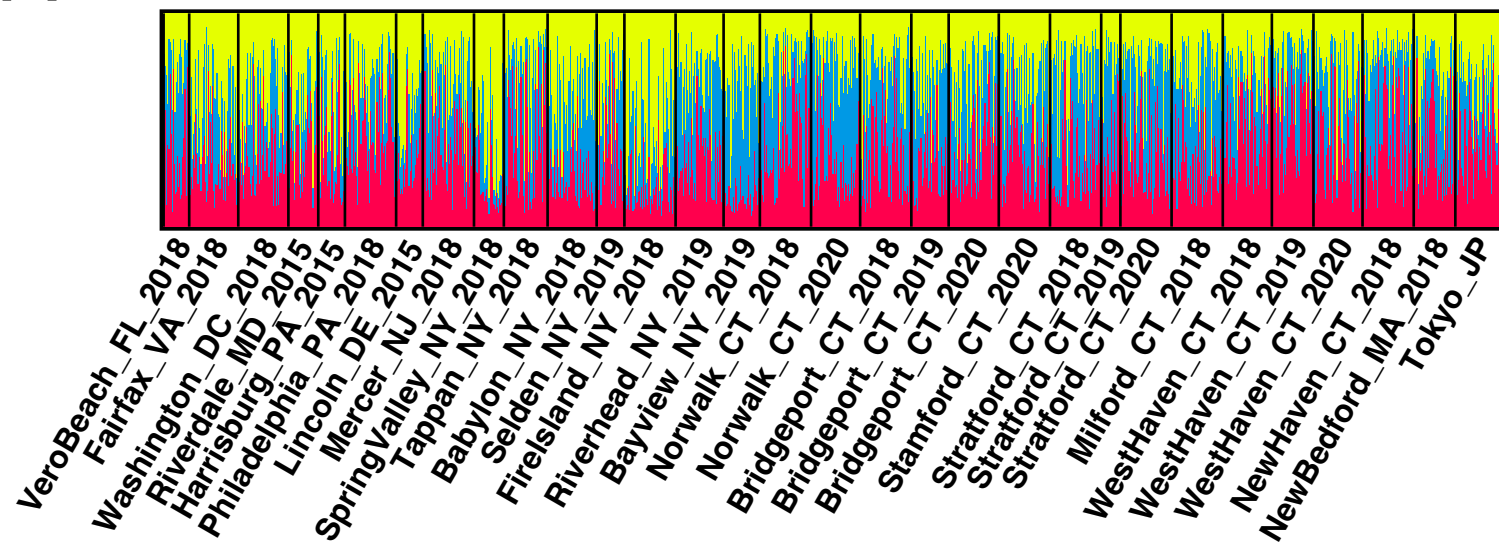

B

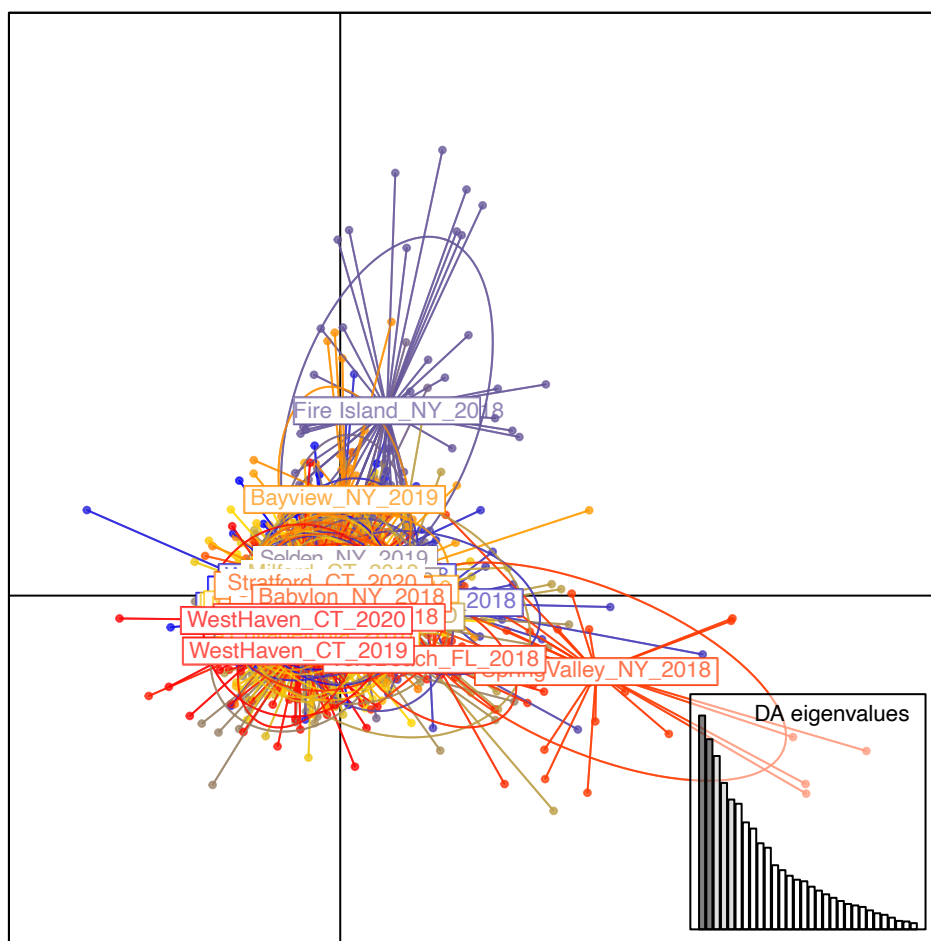

Supplement: Supplementary material 11 [file NIHMS1858977-supplement-Supplementary_material_11.pdf]

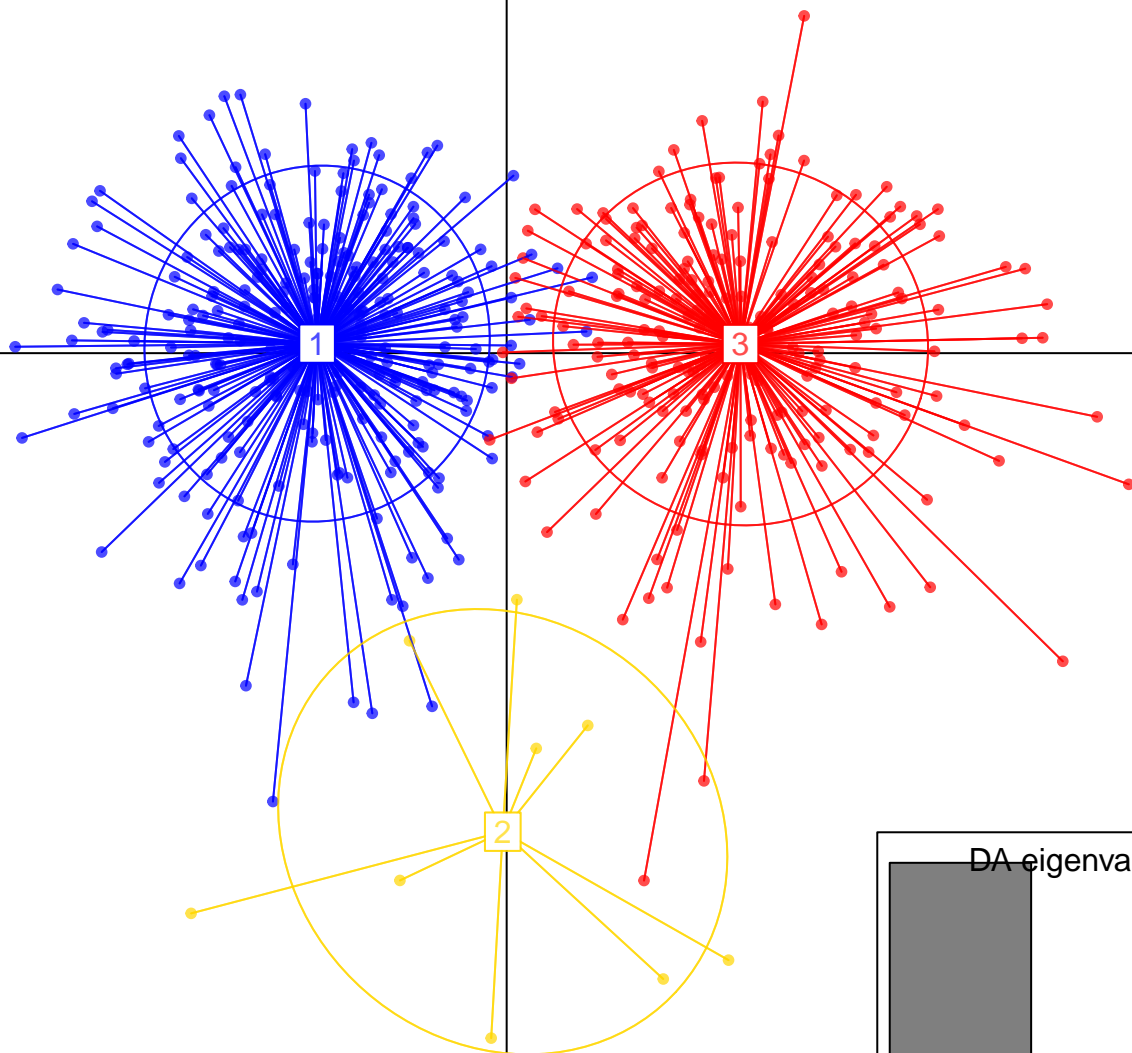

Supplement: Supplementary material 15 [file NIHMS1858977-supplement-Supplementary_material_15.pdf]

A

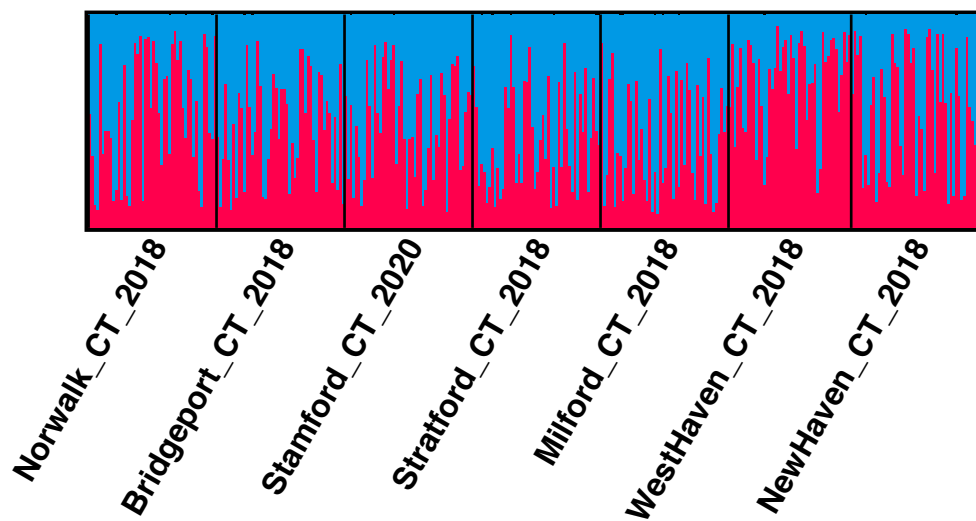

B

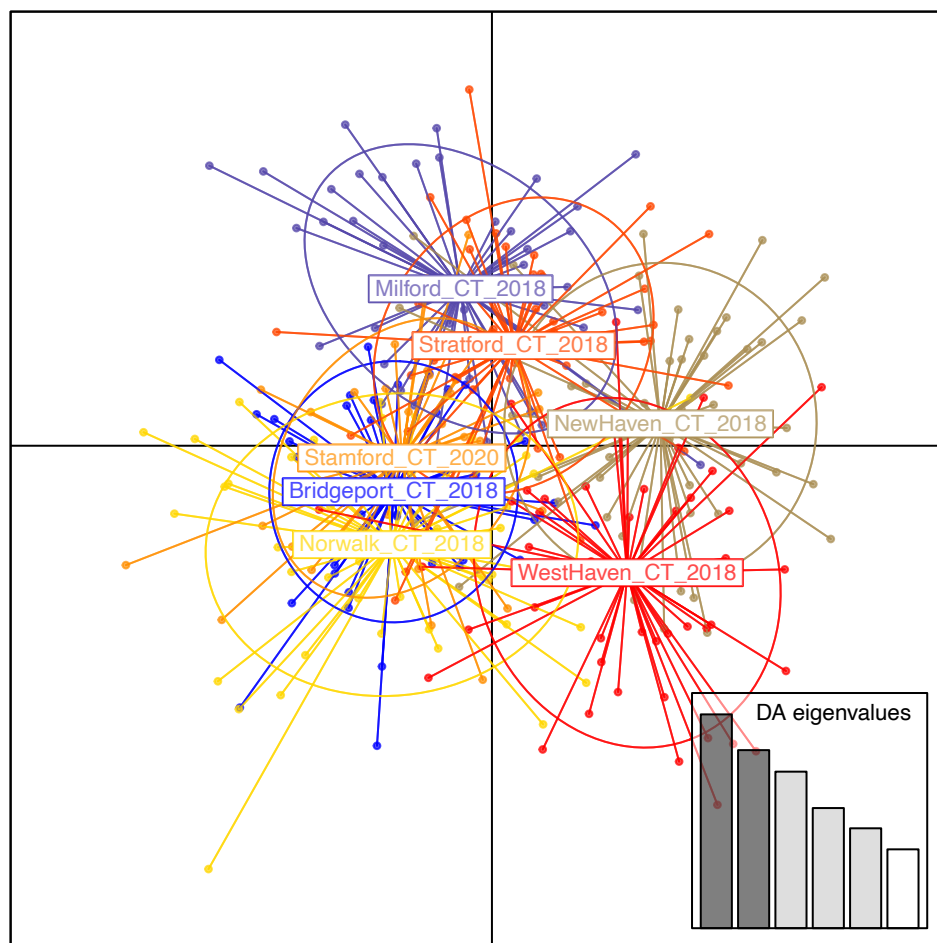

Supplement: Supplementary material 14 [file NIHMS1858977-supplement-Supplementary_material_14.pdf]

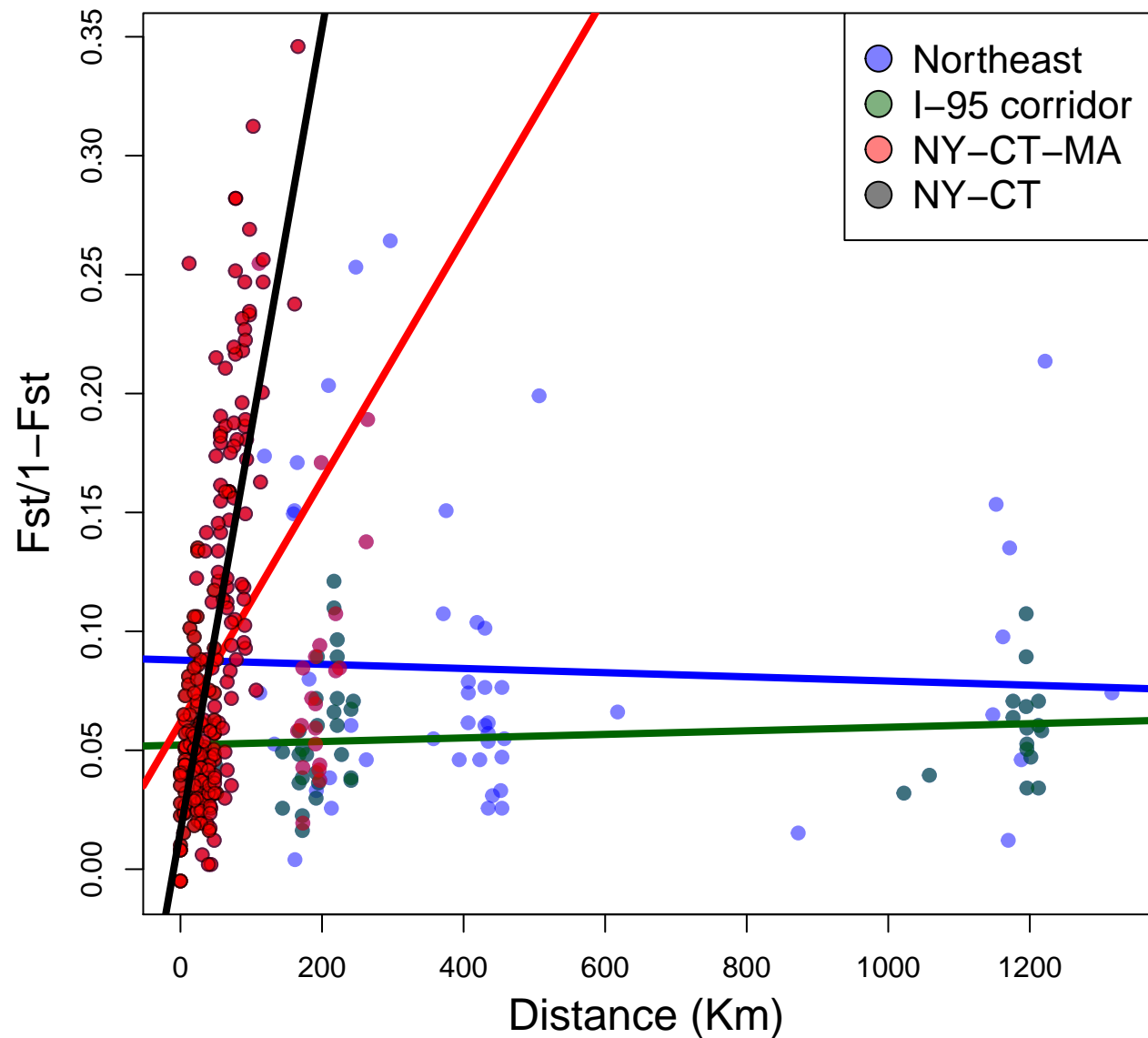

Supplement: Supplementary material 13 [file NIHMS1858977-supplement-Supplementary_material_13.pdf]
