# Supplementary material for: Population genetics of an invasive mosquito vector, Aedes albopictus in the Northeastern USA
Source: NeoBiota. Author manuscript; Available in PMC 2023 Jul 5. (PMC10321554; doi:10.3897/neobiota.78.84986)
Supplement: 1 [file NIHMS1858977-supplement-1.pdf]

Supplementary material 1

Detailed collection information

Authors: Andrea Gloria-Soria, Talya Shragai, Alexander T. Ciota, Todd B. Duval, Barry W. Alto, Ademir J. Martins, Kathleen M. Westby, Kim A. Medley, Isik Unlu, Scott R. Campbell, Malgorzata Kawalkowski, Yoshio Tsuda, Yukiko Higa, Nicholas Indelicato, Paul T. Leisnham, Adalgisa Caccone, Philip M. Armstrong

Data type: table (excel file)

Copyright notice: This dataset is made available under the Open Database License (http://opendatacommons.org/licenses/odbl/1.0/). The Open Database License (ODbL) is a license agreement intended to allow users to freely share, modify, and use this Dataset while maintaining this same freedom for others, provided that the original source and author(s) are credited.

Link: https://doi.org/10.3897/neobiota.78.84986.suppl1

Supplementary material 2

***Aedes albopictus* microsatellite primers used in this study**

Authors: Andrea Gloria-Soria, Talya Shragai, Alexander T. Ciota, Todd B. Duval, Barry W. Alto, Ademir J. Martins, Kathleen M. Westby, Kim A. Medley, Isik Unlu, Scott R. Campbell, Malgorzata Kawalkowski, Yoshio Tsuda, Yukiko Higa, Nicholas Indelicato, Paul T. Leisnham, Adalgisa Caccone, Philip M. Armstrong

Data type: table (excel file)

Copyright notice: This dataset is made available under the Open Database License (http://opendatacommons.org/licenses/odbl/1.0/). The Open Database License (ODbL) is a license agreement intended to allow users to freely share, modify, and use this Dataset while maintaining this same freedom for others, provided that the original source and author(s) are credited.

Link: https://doi.org/10.3897/neobiota.78.84986.suppl2

Supplementary material 3

Microsatellite bins used to call alleles in Geneious v. 11.1.5 (Biomatters LTD)

Authors: Andrea Gloria-Soria, Talya Shragai, Alexander T. Ciota, Todd B. Duval, Barry W. Alto, Ademir J. Martins, Kathleen M. Westby, Kim A. Medley, Isik Unlu, Scott R. Campbell, Malgorzata Kawalkowski, Yoshio Tsuda, Yukiko Higa, Nicholas Indelicato, Paul T. Leisnham, Adalgisa Caccone, Philip M. Armstrong

Data type: table (excel file)

Copyright notice: This dataset is made available under the Open Database License (http://opendatacommons.org/licenses/odbl/1.0/). The Open Database License (ODbL) is a license agreement intended to allow users to freely share, modify, and use this Dataset while maintaining this same freedom for others, provided that the original source and author(s) are credited.

Link: https://doi.org/10.3897/neobiota.78.84986.suppl3

Supplementary material 4

Allele numbers and allelic richness at 15 microsatellite loci used in this study

Authors: Andrea Gloria-Soria, Talya Shragai, Alexander T. Ciota, Todd B. Duval, Barry W. Alto, Ademir J. Martins, Kathleen M. Westby, Kim A. Medley, Isik Unlu, Scott R. Campbell, Malgorzata Kawalkowski, Yoshio Tsuda, Yukiko Higa, Nicholas Indelicato, Paul T. Leisnham, Adalgisa Caccone, Philip M. Armstrong

Data type: table (excel file)

Explanation note: Allele numbers and allelic richness at 15 microsatellite loci used in this study obtained using rarefaction to correct for unequal sample sizes (N = 30 genes) in HP-RARE (Kalinowski 2005).

Copyright notice: This dataset is made available under the Open Database License (http://opendatacommons.org/licenses/odbl/1.0/). The Open Database License (ODbL) is a license agreement intended to allow users to freely share, modify, and use this Dataset while maintaining this same freedom for others, provided that the original source and author(s) are credited.

Link: https://doi.org/10.3897/neobiota.78.84986.suppl4

Supplementary material 5

Probability of a recent bottleneck at each *Aedes albopictus* location, under the infinite allele model (IAM) and the two-phase model (TPM) with variance of 0.36

Authors: Andrea Gloria-Soria, Talya Shragai, Alexander T. Ciota, Todd B. Duval, Barry W. Alto, Ademir J. Martins, Kathleen M. Westby, Kim A. Medley, Isik Unlu, Scott R. Campbell, Malgorzata Kawalkowski, Yoshio Tsuda, Yukiko Higa, Nicholas Indelicato, Paul T. Leisnham, Adalgisa Caccone, Philip M. Armstrong

Data type: table (excel file)

Explanation note: Probability of a recent bottleneck at each *Aedes albopictus* location, under the infinite allele model (IAM) and the two-phase model (TPM) with variance of 0.36; as estimated using the software Bottleneck v. 1.2.02 (Cornuet and Luikart, 1997). The Wilcoxon sign-rank test (Luikart et al. 1998) was used to determine significance. Values in bold indicate significant differences after Bonferroni multiple test correction.

Copyright notice: This dataset is made available under the Open Database License (http://opendatacommons.org/licenses/odbl/1.0/). The Open Database License (ODbL) is a license agreement intended to allow users to freely share, modify, and use this Dataset while maintaining this same freedom for others, provided that the original source and author(s) are credited.

Link: https://doi.org/10.3897/neobiota.78.84986.suppl5

Supplementary material 6

Kinship analysis

Authors: Andrea Gloria-Soria, Talya Shragai, Alexander T. Ciota, Todd B. Duval, Barry W. Alto, Ademir J. Martins, Kathleen M. Westby, Kim A. Medley, Isik Unlu, Scott R. Campbell, Malgorzata Kawalkowski, Yoshio Tsuda, Yukiko Higa, Nicholas Indelicato, Paul T. Leisnham, Adalgisa Caccone, Philip M. Armstrong

Data type: table (excel file)

Explanation note: Summary of pedigree relationships within *Aedes albopictus* collections. Unrelated (U), half-siblings (HS), full-siblings (FS), and parent-offspring (PO), as estimated by ML-Relate (Kalinowski et al. 2006). Values in bold highlight collections with the frequency of first-degree relatives above 5%.

Copyright notice: This dataset is made available under the Open Database License (http://opendatacommons.org/licenses/odbl/1.0/). The Open Database License (ODbL) is a license agreement intended to allow users to freely share, modify, and use this Dataset while maintaining this same freedom for others, provided that the original source and author(s) are credited.

Link: https://doi.org/10.3897/neobiota.78.84986.suppl6

Supplementary material 7

Isolation by distance analyses (IBD)

Authors: Andrea Gloria-Soria, Talya Shragai, Alexander T. Ciota, Todd B. Duval, Barry W. Alto, Ademir J. Martins, Kathleen M. Westby, Kim A. Medley, Isik Unlu, Scott R. Campbell, Malgorzata Kawalkowski, Yoshio Tsuda, Yukiko Higa, Nicholas Indelicato, Paul T. Leisnham, Adalgisa Caccone, Philip M. Armstrong

Data type: table (excel file)

Explanation note: Matrices of geographic distance in meters and genetic distance as Fst. Results from Mantel tests for *Aedes albopictus* in the northeastern USA; the I-95 interstate corridor from Virginia (VA) to CT; the northeastern expansion front (New York, Connecticut, Massachusetts); and through Connecticut and New York.

Copyright notice: This dataset is made available under the Open Database License (http://opendatacommons.org/licenses/odbl/1.0/). The Open Database License (ODbL) is a license agreement intended to allow users to freely share, modify, and use this Dataset while maintaining this same freedom for others, provided that the original source and author(s) are credited.

Link: https://doi.org/10.3897/neobiota.78.84986.suppl7

Supplementary material 8

Genetic clusters inferred from all Connecticut collections using discriminant analysis of principal components in the ADEGENET package

Authors: Andrea Gloria-Soria, Talya Shragai, Alexander T. Ciota, Todd B. Duval, Barry W. Alto, Ademir J. Martins, Kathleen M. Westby, Kim A. Medley, Isik Unlu, Scott R. Campbell, Malgorzata Kawalkowski, Yoshio Tsuda, Yukiko Higa, Nicholas Indelicato, Paul T. Leisnham, Adalgisa Caccone, Philip M. Armstrong

Data type: table (excel file)

Explanation note: Genetic clusters inferred from all Connecticut collections using discriminant analysis of principal components in the ADEGENET package (Jombart 2008) in R v. 3.2.2. (R Core Team 2018).

Copyright notice: This dataset is made available under the Open Database License (http://opendatacommons.org/licenses/odbl/1.0/). The Open Database License (ODbL) is a license agreement intended to allow users to freely share, modify, and use this Dataset while maintaining this same freedom for others, provided that the original source and author(s) are credited.

Link: https://doi.org/10.3897/neobiota.78.84986.suppl8

Supplementary material 9

Latitude of each northeastern USA *Aedes albopictus* location

Authors: Andrea Gloria-Soria, Talya Shragai, Alexander T. Ciota, Todd B. Duval, Barry W. Alto, Ademir J. Martins, Kathleen M. Westby, Kim A. Medley, Isik Unlu, Scott R. Campbell, Malgorzata Kawalkowski, Yoshio Tsuda, Yukiko Higa, Nicholas Indelicato, Paul T. Leisnham, Adalgisa Caccone, Philip M. Armstrong

Data type: figure (pdf file)

Explanation note: Latitude of each northeastern USA *Aedes albopictus* location plotted against A its observed heterozygosity (Ho) and B allelic richness estimated by rarefaction (N = 30). Linear regression in R v. 3.2.2. (R Core Team 2018) indicates a correlation between latitude and Ho (adjusted R2 = 0.13, F(1,27), p = 0.03) but not between latitude and allelic richness (adjusted R2 = 0.04, F(1,27), p = 0.15).

Copyright notice: This dataset is made available under the Open Database License (http://opendatacommons.org/licenses/odbl/1.0/). The Open Database License (ODbL) is a license agreement intended to allow users to freely share, modify, and use this Dataset while maintaining this same freedom for others, provided that the original source and author(s) are credited.

Link: https://doi.org/10.3897/neobiota.78.84986.suppl9

Supplementary material 10

Estimates of effective population size based of Connecticut populations obtained with NeEstimator (Do et al. 2014)

Authors: Andrea Gloria-Soria, Talya Shragai, Alexander T. Ciota, Todd B. Duval, Barry W. Alto, Ademir J. Martins, Kathleen M. Westby, Kim A. Medley, Isik Unlu, Scott R. Campbell, Malgorzata Kawalkowski, Yoshio Tsuda, Yukiko Higa, Nicholas Indelicato, Paul T. Leisnham, Adalgisa Caccone, Philip M. Armstrong

Data type: figure (pdf file)

Explanation note: Estimates of effective population size based of Connecticut populations obtained with NeEstimator (Do et al. 2014) using A population pairs using the two-sample Waples (1989) method and three options for computing the standardized variance in allele frequency, F [Fe (Nei and Tajima 1981); Fk (Pollak 1983); and Fs (Jorde and Ryman 2007)]; and B a single population sample using the bias-corrected version of the linkage disequilibrium method Waples and Do (2008). Mean effective population size estimates (Ne), 95% confidence intervals (CI) are displayed by locality. The average Ne across all estimates is displayed with a dashed (harmonic mean) and dotted (arithmetic mean) horizontal lines. Note that the arithmetic mean of B is missing as it falls outside the plotted area (Ne = 2,337.5).

Copyright notice: This dataset is made available under the Open Database License (http://opendatacommons.org/licenses/odbl/1.0/). The Open Database License (ODbL) is a license agreement intended to allow users to freely share, modify, and use this Dataset while maintaining this same freedom for others, provided that the original source and author(s) are credited.

Link: https://doi.org/10.3897/neobiota.78.84986.suppl10

Supplementary material 11

Population structure of *Aedes albopictus* from the United States and Japan based on 15 microsatellite markers

Authors: Andrea Gloria-Soria, Talya Shragai, Alexander T. Ciota, Todd B. Duval, Barry W. Alto, Ademir J. Martins, Kathleen M. Westby, Kim A. Medley, Isik Unlu, Scott R. Campbell, Malgorzata Kawalkowski, Yoshio Tsuda, Yukiko Higa, Nicholas Indelicato, Paul T. Leisnham, Adalgisa Caccone, Philip M. Armstrong

Data type: figure (pdf file)

Explanation note: A STRUCTURE plot with each individual represented by a vertical bar. The height of each bar is the probability of assignment to each of K = 3 genetic clusters (indicated by different colors). B Discriminant analysis of principal components (DAPC).

Copyright notice: This dataset is made available under the Open Database License (http://opendatacommons.org/licenses/odbl/1.0/). The Open Database License (ODbL) is a license agreement intended to allow users to freely share, modify, and use this Dataset while maintaining this same freedom for others, provided that the original source and author(s) are credited.

Link: https://doi.org/10.3897/neobiota.78.84986.suppl11

Supplementary material 12

Population structure of *Aedes albopictus* at the United States northeastern invasion front (New York, Connecticut, Massachusetts) based on 15 microsatellite markers

Authors: Andrea Gloria-Soria, Talya Shragai, Alexander T. Ciota, Todd B. Duval, Barry W. Alto, Ademir J. Martins, Kathleen M. Westby, Kim A. Medley, Isik Unlu, Scott R. Campbell, Malgorzata Kawalkowski, Yoshio Tsuda, Yukiko Higa, Nicholas Indelicato, Paul T. Leisnham, Adalgisa Caccone, Philip M. Armstrong

Data type: figure (pdf file)

Explanation note: Population structure of *Aedes albopictus* at the United States northeastern invasion front (New York, Connecticut, Massachusetts) based on 15 microsatellite markers. A Discriminant analysis of principal components (DAPC) and B STRUCTURE plot with each individual represented by a vertical bar. The height of each bar is the probability of assignment to each of K = 3 and K = 6 genetic clusters (indicated by different colors).

Copyright notice: This dataset is made available under the Open Database License (http://opendatacommons.org/licenses/odbl/1.0/). The Open Database License (ODbL) is a license agreement intended to allow users to freely share, modify, and use this Dataset while maintaining this same freedom for others, provided that the original source and author(s) are credited.

Link: https://doi.org/10.3897/neobiota.78.84986.suppl12

Supplementary material 13

Geographic genetic differentiation (IBD: isolation by distance) across the Northeast USA

Authors: Andrea Gloria-Soria, Talya Shragai, Alexander T. Ciota, Todd B. Duval, Barry W. Alto, Ademir J. Martins, Kathleen M. Westby, Kim A. Medley, Isik Unlu, Scott R. Campbell, Malgorzata Kawalkowski, Yoshio Tsuda, Yukiko Higa, Nicholas Indelicato, Paul T. Leisnham, Adalgisa Caccone, Philip M. Armstrong

Data type: figure (pdf file)

Explanation note: Genetic distance is given as the linearized Fst [Fst/(1/Fst)] and geographic distance is provided in kilometers (Km).

Copyright notice: This dataset is made available under the Open Database License (http://opendatacommons.org/licenses/odbl/1.0/). The Open Database License (ODbL) is a license agreement intended to allow users to freely share, modify, and use this Dataset while maintaining this same freedom for others, provided that the original source and author(s) are credited.

Link: https://doi.org/10.3897/neobiota.78.84986.suppl13

Supplementary material 14

Population structure on *Aedes albopictus* samples from all Connecticut samples (no temporal series) based on 15 microsatellite markers

Authors: Andrea Gloria-Soria, Talya Shragai, Alexander T. Ciota, Todd B. Duval, Barry W. Alto, Ademir J. Martins, Kathleen M. Westby, Kim A. Medley, Isik Unlu, Scott R. Campbell, Malgorzata Kawalkowski, Yoshio Tsuda, Yukiko Higa, Nicholas Indelicato, Paul T. Leisnham, Adalgisa Caccone, Philip M. Armstrong

Data type: figure (pdf file)

Explanation note: A STRUCTURE plot with each individual represented by a vertical bar. The height of each bar is the probability of assignment to each of K = 3 genetic clusters (indicated by different colors). B Discriminant analysis of principal components (DAPC).

Copyright notice: This dataset is made available under the Open Database License (http://opendatacommons.org/licenses/odbl/1.0/). The Open Database License (ODbL) is a license agreement intended to allow users to freely share, modify, and use this Dataset while maintaining this same freedom for others, provided that the original source and author(s) are credited.

Link: https://doi.org/10.3897/neobiota.78.84986.suppl14

Supplementary material 15

Inferred genetic clusters from *Aedes albopictus* of the Connecticut temporal series

Authors: Andrea Gloria-Soria, Talya Shragai, Alexander T. Ciota, Todd B. Duval, Barry W. Alto, Ademir J. Martins, Kathleen M. Westby, Kim A. Medley, Isik Unlu, Scott R. Campbell, Malgorzata Kawalkowski, Yoshio Tsuda, Yukiko Higa, Nicholas Indelicato, Paul T. Leisnham, Adalgisa Caccone, Philip M. Armstrong

Data type: figure (pdf file)

Explanation note: Inferred genetic clusters from *Aedes albopictus* of the Connecticut temporal series using Discriminant Analysis of Principal Components in ADEGENET (Jombart 2008)

Copyright notice: This dataset is made available under the Open Database License (http://opendatacommons.org/licenses/odbl/1.0/). The Open Database License (ODbL) is a license agreement intended to allow users to freely share, modify, and use this Dataset while maintaining this same freedom for others, provided that the original source and author(s) are credited.

Link: https://doi.org/10.3897/neobiota.78.84986.suppl15
